# Perceptions and uptake of health insurance for maternal care in rural Kenya: a cross sectional study

**DOI:** 10.11604/pamj.2016.23.125.8936

**Published:** 2016-03-25

**Authors:** Jackson Michuki Maina, Peter Kithuka, Samuel Tororei

**Affiliations:** 1 School of Public Health, Moi University, Kenya; 2 Department of Health management and informatics, Kenyatta University, Kenya

**Keywords:** Health insurance, antenatal care, insurance uptake

## Abstract

**Introduction:**

In Kenya, maternal and child health accounts for a large proportion of the expenditures made towards healthcare. It is estimated that one in every five Kenyans has some form of health insurance. Availability of health insurance may protect families from catastrophic spending on health. The study intended to determine the factors affecting the uptake of health insurance among pregnant women in a rural Kenyan district.

**Methods:**

This was cross-sectional study that sampled 139 pregnant women attending the antenatal clinic at a level 5 hospital in a Kenyan district. The information was collected through a pretested interview schedule.

**Results:**

The median age of the study participants was 28 years. Out of the 139 respondents, 86(62%) planned to pay for their deliveries through insurance. There was a significant relationship between insurance uptake and marital status Adjusted odds ratio (AOR) 6.4(1.4-28.8). Those with tertiary education were more likely to take up insurance AOR 5.1 (1.3-19.2). Knowing the benefits of insurance and the limits the insurance would settle in claims was associated with an increase in the uptake of insurance AOR 7.6(2.3-25.1), AOR 6.4(1.5-28.3) respectively. Monthly income and number of children did not affect insurance uptake.

**Results:**

Being married, tertiary education and having some knowledge on how insurance premiums are paid are associated with uptake of medical insurance. Information generated from this study if utilized will bring a better understanding as to why insurance coverage may be low and may provide a basis for policy changes among the insurance companies to increase the uptake.

## Introduction

According to the 2014 demographic and health survey, the maternal mortality ratio stands at 362 per 100,000 live births [1]. Although this dropped from the previous ratio of 520 per 100000 live births, many agree the ratio is still unacceptably high. Home deliveries and delivery by unskilled personnel have been thought to contribute to these high ratios. To this end, there has been a push to encourage pregnant women to visit health facilities for antenatal care and to deliver in health facilities. The national survey indicates that about 61% of women delivered in a health facility between 2010 and 2014 [1]. Appropriate health financing measures could hold the key to improving maternal health in Kenya and other countries by increasing access to and utilization of health services [2]. However, paying for health care remains a major challenge in Kenya and most African countries [3, 4]. In Kenya, the government contributes 41% of the total health expenditure with households “out of pocket” contributing to about 30% of the expenditure [5]. Paying for healthcare out of pocket, may push households into poverty with 6-10% of households reporting catastrophic spending on health [6]. This leads families to seek alternate sources of financing including health insurance and community financing schemes. The use of supply side financing initiatives may limit patient choice of health facility while also not encouraging the delivery of quality services. Contributory mechanisms such as health insurance provide a means for patients to exercise some choice over facility and send appropriate signals to encourage quality of care at health facilities by giving consumers the power to choose. Insurance coverage has been on the increase in the last decade with about 20% of Kenyans having some form of health insurance coverage up from around 10% in 2006 [7]. In terms of insurance coverage, the National Hospital Insurance Fund (NHIF) and private insurance covered 88.4 and 9.4 percent respectively. Community-based and other forms of insurance coverage covered 1.3 percent and 1.0 percent, respectively [6]. The NHIF which is a government operated insurance which covers members in different health quantiles is the largest insurance provider in the rural areas. It is compulsory for those in the formal sector but for those in the informal sector and retirees it is voluntary. The fund′s core mandate is to provide medical insurance cover to all its members and their declared dependents (spouse and children). The NHIF membership is open to all Kenyans who have attained the age of 18 years and have a monthly income of more than $10 [8]. At the time of this study, NHIF was principally covering inpatient cost and had no outpatient benefits. In Kenya, the highest insurance coverage is among men and children under 5 and adults over 65 years and those in urban areas [9]. There have been several attempts to explore the factors that increase uptake of insurance with an attempt to leverage on those to increase the coverage of health insurance. Insurance uptake has been found to increase as one advance in age. This may be due to an increase in purchasing power [10]. Subsequently women of child bearing age (18-45 years) are excluded from insurance and this affects more so those in the rural areas [6]. Those in formal employment are more likely to have some form of medical insurance unlike those in the informal sector and those who are unemployed [11, 12]. Other factors like, marital status, educational status, profession and household income all have significant impact on uptake of insurance [13]. There have been several qualitative assessments of insurance uptake to understand the attitudes and perceptions towards health insurance, Some still think insurance is for those in the formal sector and others do not understand how they would pay for a service that they may not use [14]. In areas where little information about the insurance companies is available, some people may be opposed to giving money to insurance companies in the fear that the money will be misappropriated and they may not get the services paid for [15]. In Kenya, health services are provided in 6 levels from level one which is the lowest level of care in the community by community health workers to level six which are referral hospitals. Antenatal care, maternity services and vaccination are provided from level two (dispensaries) to level 6 [16]. These services were mainly under the central government, but in 2010 a new constitution devolved the administration into 47 counties [17]. These counties are now responsible for the management of level one to level five facilities and the central government manages the level six facilities. With this basic understanding of the current health service and financing system, we sought to understand the perceptions and practices of women of in child bearing age particularly in rural areas health towards insurance. This study aimed to determine the factors that affect the uptake of the health insurance among pregnant women attending an antenatal clinic (ANC) in a level five facility in rural Kenya. We chose to recruit pregnant women at ANC because these visits were not payable by insurance hence avoiding selection bias, and these women would subsequently be hospitalized were for their upcoming deliveries and hence would need to make a choice on the mode of payment for their delivery. Our choice of a level five facility was based on the fact that there are specialized obstetric services offered at this level hence more women were likely to visit and the fact that level five facilities cover a large catchment hence a wide mix of patients of diverse age and socioeconomic status and in theory provides a balance of supply and demand.

## Methods

This was a cross sectional study conducted at the antenatal clinic of Embu level five hospitals. Embu County is one of the 47 counties in Kenya. It is divided in four sub counties and has a total population of 543,221. The level five hospitals serve as a referral institution for the county. The county has two level four facilities, two level three facilities and more than 60 level one facilities. Embu district where the main level five hospitals are located has about 31 facilities which are mainly level 2 and level 3 facilities. This is in addition to private clinics. Some of these facilities have small ANC but the main ANC is at Embu level five hospitals. The ratio of hospital beds to population in Embu is 1:522 while doctor/patient ratio is 1:10,482 [18]. The study was carried out in February 2012, five days a week (Monday - Friday), which are the clinic days. Those eligible to participate in this study were pregnant women visiting the Embu level five hospitals for their routine ANC follow up and those who had come in to seek treatment during pregnancy. Women who were not pregnant and those who declined to give consent for the study were excluded. Based on a sample size calculation for discrete data assuming an insurance coverage of 10% we calculated a required sample size of 139 [19]. Using the available records of the previous year, the hospital recorded an average of 486 women visiting the clinic every month [20]. Thus using systematic sampling we used the attendance register to pick every third woman registered by the clerk on arrival to the clinic every day clinic day of the study month. Questionnaires with structured closed questions were administered to the eligible women in the antenatal clinic by the investigator and trained assistants. The data collection tool was translated into Kiswahili to accommodate respondents whose mastery of English was poor. Once the data were collected, the investigator went through the questionnaires to ensure they were complete before the participant left and for any missing fields the participant was asked for their response. The data collected through the questionnaires were coded, entered and analyzed using the Statistical Package for the Social Sciences (SPSS) windows version 17.0. Descriptive statistics were used to describe the main variables on socio demographic features, knowledge, attitude and practices. The proportion of those who would take up insurance was described as the main outcome variable. Measures of association were calculated using chi square and significant factors were then subjected to a logistic regression model This research was approved by the research and ethics committee of Moi University Kenya before commencement: IREC Ref. 2011/136 approval number 000747. Written informed consent was obtained from all the study participants before participating in the study. The consent was available in English and in Swahili for those who did not understand English.

## Results

A total of 139 participants were interviewed. The median age was 28 years, (Interquartile range (IQR) 18-43). Majority (75%) were married and 37% of the study participants were in their first pregnancy. 86(62%) of the women recruited in the study indicated that they would be paying for their deliveries or hospitalizations using medical insurance. NHIF was the preferred choice in all the cases. 23(17%) of the women would pay out of pocket and another 17% would request for assistance from relatives. The remainder (5%) had no idea of how they will pay for the delivery.

### Socio-demographic factors

Marital status, Odds Ratio (OR) 2.4 (1.4 -5.8), Tertiary education level OR 7.6(2.7-21.1) and a monthly income of $55 OR 4.6(1.8-11.5) were associated with uptake of insurance. Table 1 summarizes the various socio demographic factors in relation to insurance uptake.

**Table 1:** Baseline characteristics affecting insurance uptake

Variable	Study participants (%) N= 139	Have Insurance (%) N = 86	OR (95% CI)	P value
No of children				
2 or more	47 (34)	30 (35)	1.1 (0.6-2.4)	0.74
0-1	92 (66)	56(65)	Ref	
Marital Status				
Married	104 (75)	70 (81)	2.4(1.1-5.4)	0.03
Single or others	35 (25)	16(19)	Ref	
Age in years				
Under 24	36 (26)	18 (21)	0.7(0.2-2.0)	0.48
25-29	49 (35)	30 (35)	1(0.4-3)	0.92
30-35	34 (24)	26 (30)	2.2(0.7-7)	0.20
Over 35 years	20 (14)	12 (14)	Ref	
Highest attained education level				
Tertiary	48 (35)	41 (48)	7.6(2.7-21.1)	< 0.01
Secondary	52 (37)	28 (33)	1.5(0.7-3.5)	0.36
Primary	39 (28)	17 (20)	Ref	
Employment Status				
Employed	77 (55)	57(34)	3.2(1.5-7.1)	<0.01
Unemployed	62 (45)	29(47)		
Average Monthly /income(USD)				
< 55	18 (13)	9 (10)	1.1(0-.4-3.2)	0.81
55-170	40 (29)	32 (37)	4.6(1.8-11.5)	< 0.01
>170	19 (14)	16 (19)	6.1(1.6-23.1)	< 0.01
Unemployed	62 (45)	29 (34)	Ref	

### Knowledge on insurance

Participants’ knowledge was probed using 8 questions. About 86(62%) of respondents indicated they would pay for their delivery through insurance, with NHIF being the most preferred insurance. Knowing health insurance to be beneficial OR 10.2 (4.5-23.3), knowing how insurance premiums and claims relate OR 9.1(3.7-22.6) and knowing the limits in claims one is entitled to OR 7.3(2.4-21.1) were associated with increased insurance uptake.

### Attitude towards insurance

Participants’ attitude was probed using four questions. In the case of hospitalization, 69(80%) of those with insurance felt they still had enough money to pay out of pocket. Those who felt that amounts paid to insurance was not large enough to affect the meeting of basic needs OR 3.4(1.5-7.5) and the feeling that is was an wise investment to have medical insurance OR 1.5(0.7-3.3) were associated with an increase insurance uptake.

### Practices among the study participants

Practices were assessed using 5 questions. 30% of the participants reported a previous hospitalization. 9% of those who had been hospitalized had paid for their care using medical insurance. From the respondents, having been hospitalized in the previous year OR 0.9(0.4-1.8), one was more likely to take up insurance. 58(67%) of those who has insurance felt there were more likely to seek medical care. The summary of the factors associated with an increase in the insurance uptake are summarized in Table 2 below.

**Table 2:** Knowledge, attitudes and practices affecting insurance uptake

Variable	Have Insurance N = 86 n (%)	Odds Ratio (95% CI)	P Value
**Knowledge**			
Know health insurance to be beneficial	74(86)	10.2(4.5-23.3)	<0.01
Know Relationship between premiums and claims	50(58)	9.1(3.7-22.6)	<0.01
Know Amounts paid for claims	32(37)	7.3(2.4-22.1)	<0.01
**Attitudes**			
Enough money in savings to pay for medical bills	69(80)	3.9(1.8-8.4)	<0.01
Amounts paid to insurance not substantial to affect meeting the basic needs	72 (84)	3.4 (1.5-7.5)	<0.01
Paying insurance as a wise investment on health	67(78)	1.5(0.7-3.3)	0.29
**Practice**			
Hospitalization in preceding year	25(29)	0.9(0.4-1.8)	0.85
Influence of hospitalization to take up insurance	20(23)	2.0(0.8-5.1)	0.15
More likely to seek medical care if having insurance	58(67)	1.9(0.9-3.7)	0.19

### Multivariable analysis

The factors that were significantly related to insurance uptake were; marital status, education level, monthly income, knowledge that health insurance to be beneficial, Knowing the relationship between premiums and claims, Knowing amounts paid for claims by insurance, participants feeling they have enough money in savings to pay for medical bills and feeling that the amounts paid to insurance are not substantial to affect them meeting their basic needs. These were considered for multivariable analysis. There was a significant relationship between insurance uptake and marital status AOR 6.4(1.4-28.8). Those with tertiary education were more likely to take up insurance AOR 5.1(1.3-19.2). Knowing the benefits of insurance and the limits the insurance would settle in claims was associated with an increase in the uptake of insurance AOR 7.6(2.3-25.1), AOR 6.4(1.5-28.3) respectively. Monthly income did not seem to affect insurance uptake. Employment status was excluded from the multivariate analysis due to co linearity. Table 3 summarizes the multivariable analysis.

**Table 3:** Multivariable analysis

Variable	b	S.Eb.	df	Sig.	Adjusted OR (95% CI)
**Marital status**					
Married	1.849	0.771	1	**0.02**	6.4 (1.4-28.8)
Single or others	Reference				
**Education level**					
Secondary	0.049	0.602	1	0.94	1.1 (0.3-3.4)
Tertiary or others	1.636	0.672	1	**0.02**	5.1(1.3-19.2)
Primary	Reference				
**Monthly income**					
< $ 55	0.247	0.713	1	0.73	1.3(0.3-5.2)
$ 55-170	0.993	0.712	1	0.16	2.7(0.7-10.9)
>$ 170	1.061	0.648	1	0.10	2.9(0.8-10.3)
Not Employed	Reference				
**Know health insurance to be beneficial**					
Yes	2.030	0.610	1	**<0.01**	7.6(2.3-25.1)
No or don't know	Reference				
**Know Relationship between premiums and claims**					
No idea	0.051	0.827	1	0.95	1.1(0.2-5.3)
Have an idea on the relationship	Reference				
**Know Amounts paid for claims by insurance**					
Have an idea	1.860	0.757	1	**0.01**	6.4(1.5-28.3)
Have no idea	Reference				
**Enough money in savings to pay for medical bills**					
Yes	0.998	0.888	1	0.26	2.7(0.5-15.5)
No	Reference				
**Amounts paid to insurance not substantial to affect meeting the basic needs**					
Yes	0.222	0.980	1	0.82	1.2(0.2-8.5)
No	Reference				

## Discussion

This study aimed to determine the factors that affect the uptake of the health insurance among pregnant women attending an antenatal clinic (ANC) in a level five facility in rural Kenya. In the study, uptake of insurance was at 62%. This is much higher than the current national uptake of around 20% [6]. Being married, having tertiary education and knowledge of the insurance benefits was associated with an increase in uptake. Other studies have also shown than more educated women were more likely to take up medical insurance [10]. Education is a factor that also improves the health seeking behaviour and hence insurance uptake [21]. The increased uptake among married women has been confirmed in other studies [22, 23]. Some of the hypothesized reasons are that having financial support from a spouse increases the opportunities for access of health insurance coverage as a result of increase income, another reason may be the fact that some employers provide cover for spouses and children and hence one can be insured through the spouses insurance cover [10]. In this study, the numbers of those employed and unemployed were almost similar, however employment was found to be collinear and hence was excluded in the analysis for its relation to insurance uptake. This differs from previous studies that have shown employment to be a major contributor to insurance uptake [11, 12, 24] in an effort to increase insurance coverage, there is a push to have insurance schemes for those in the informal sector through microfinance schemes [25]. Although household size and household income were not significant factors that affect uptake, household size in previous studies has been attributed as a major factor that affects uptake of insurance [26]. Once a household has many children, the resources are strained and thus find it hard to put money aside to pay for medical insurance. Several studies in Africa has shown that households with higher income were more likely to take up insurance [27, 28].

### Knowledge on Insurance

From the discussion on knowledge on insurance majority agreed that health insurance is beneficial. These findings are in keeping with previous studies among Kenyan communities who also agreed that it is important to have a form of health insurance to cater for medical expenses especially in cases of emergencies to reduced catastrophic spending on health [24]. The knowledge level is much higher compared to neighboring countries in the region [26]. This may be explained by the fact that most of the study respondents had attained at least secondary education and hence had some form of education which increased knowledge on current issues like medical insurance. The knowledge on how insurance works is also comparably low in the region as demonstrated by Asia et al in Tanzania [29].

### Attitudes and practices towards insurance

In this study most women felt they had enough money to cater for the medical bills. Previous studies have shown income to be a major contributor to health seeking behaviour and insurance uptake [30]. The effect of catastrophic financial effects was not anticipated in this group as they felt that the paying of insurance would have a minimal effect on the ability to cater for the basic needs. This may be due to the fact that most women sampled seemed to have a source of income. As in many areas of the world, having insurance may have one seeking health care more frequently [6]. Although we did not asses this directly, those with insurance indicated they were likely to visit health facilities; the concept of moral hazard in health insurance may lead to a strain in the health services in areas with high insurance as many people will seek care even though they are not sick [31]. Limitations The main limitations of this study is that it relied on the participants responses and hence not in a position to verify their marital status, education level or income level. Some of the questions were prone to recall bias. To counter this bias, we attempted to seek clarification with each participant during the interview process whenever the investigators noted the information was not consistent. The other limitation is that these findings may not be generalizable to the whole population as we did not sample any men hence a similar study in men would shed more light into the real situation in the region.

## Conclusion

This study highlights some of the attitudes and perceptions towards health insurance in a rural region where coverage is still relatively low. We conclude that in this area, being married, having tertiary education and having some knowledge on how insurance premiums are paid are associated with uptake of health insurance. As we strive towards provision on universal care, we note that insurance coverage is still low, there is need to offer alternative forms of health insurance, to offer people opportunities to interact with the insurance providers to ask questions and clarify various aspects of the insurance. We also recommend that future studies on insurance uptake should include qualitative methods of data collection to adequately capture the attitudes and feelings of those who are participating and also carry out similar work in men to provide and cleared picture of the situation.

### What is known about this topic

- Medical insurance coverage is low in the region.
- The more educated one is the more likely they are to take up insurance.

### What this study adds

- Education to at least tertiary level is what affects insurance uptake.
- Nation health insurance (NHIF) is the preferred insurance provider to most.
- Income and household size may not affect insurance uptake.
